# The assessment of self-harm as a window of opportunity for addressing domestic abuse: invited editorial on Knipe et al

**DOI:** 10.1192/bjo.2025.46

**Published:** 2025-05-13

**Authors:** Vishal Bhavsar, Anne M. Doherty

**Affiliations:** King’s Women’s Mental Health, Institute of Psychiatry, Psychology and Neuroscience, King’s College London, London, UK; Southwark Community Forensic Service, South London and Maudsley NHS Foundation Trust, London, UK; Department of Psychiatry, School of Medicine, University College Dublin, Dublin, Ireland; Department of Adult Psychiatry, Mater Misericordiae University Hospital, Dublin, Ireland

**Keywords:** Self-harm, liaison psychiatry, qualitative research, general adult psychiatry, forensic psychiatry

## Abstract

Domestic abuse harms children and families. Self-harm is associated with exposure to and perpetration of domestic abuse, but research on health service responses to self-harm in the context of domestic abuse is limited. We discuss recent work examining the response of mental health professionals to domestic abuse in the emergency department by Knipe and colleagues. Thematic analysis of interviews with 15 mental health professionals working in consultation and liaison settings helped to construct themes including a fear of deeper exploration and tensions between identification and response (‘between knowing and acting’). The paper raises important issues for quality improvement in responses to self-harm in liaison settings, including balancing time and resources across different management needs (including domestic abuse response) and professional perceptions of their own actions in clinical settings, such as acknowledging harmful behaviour. The paper demonstrates opportunities for strengthening responses to domestic abuse in professional training.


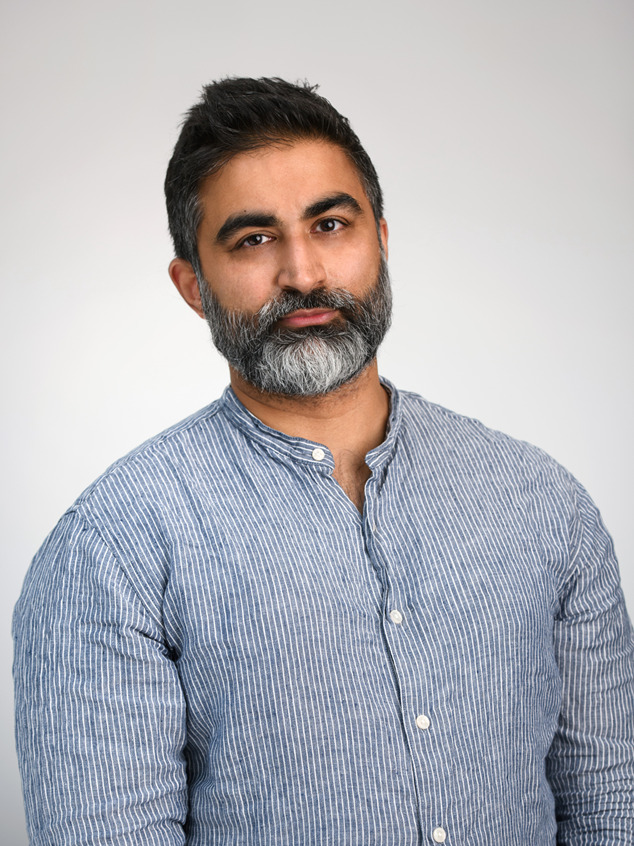
Domestic abuse, defined as harmful behaviour perpetrated towards an adult partner, ex-partner or family member, is a significant cause of mortality through homicide, especially in women.^1^ Exposure to domestic abuse is prevalent before suicide, with one-third of people who die by suicide having experienced this form of violence.^2^ Accordingly, there is strong evidence for association between experiencing domestic abuse as a victim and suicidal ideation and self-harming behaviour.^3^ Self-harm is strongly related to death by suicide: in a meta-analysis of 117 studies, 3.9% of individuals with self-harm died by suicide within 5 years.^4^ Self-harm presentations to the emergency department represent an important opportunity for suicide prevention, for example by addressing psychosocial risk factors for suicide, including substance use, housing adversity and domestic abuse. While there has been research on the identification and management of domestic abuse in health settings^5^ including mental health services,^6^ there has been limited focus on emergency departments: a scoping review of domestic abuse responses in emergency departments in 2017 found 30 studies.^7^

Research work to understand what factors shape *professional responses* to self-harm, as opposed to the factors that might *cause a person to self-harm*, could be a fruitful parallel avenue of enquiry to improving the quality and safety of care. In their recently published paper in this journal, Knipe and colleagues^8^ report a thematic analysis of interviews with 15 health professionals working in consultation-liaison psychiatry teams on the identification and assessment of domestic abuse in people who present following self-harm. The authors found that professionals acknowledged the importance of detecting and assessing the impact of domestic abuse during psychiatric consultations in the emergency department but identified barriers to doing so. There were procedural barriers and those related to resources – concerns that enquiring about domestic abuse might be too time-consuming to be undertaken during an already lengthy assessment.^9^ Professionals reflected that their own enquiry about domestic abuse was sporadic and tended to be driven by concerns about the safety of children, while certain groups, such as men and older people, were less likely to be asked whether they had experienced domestic abuse. The other barriers were caused by feeling ill-equipped to address the issues that might arise during the assessment: concern about ‘delving deeper’ and causing distress to the patient, along with the despondency experienced by the clinician when the patient returns to an abusive partner.

The findings of this paper highlight the response to domestic abuse in the emergency department as an opportunity for mental health service improvement. In particular, the economic and resource impact of initiatives to improve the assessment of domestic abuse in a consultation-liaison psychiatry setting is unknown. Research on the factors that shape disclosure and reporting of domestic abuse to professionals tends to recommend training and guideline improvement to encourage more frequent and effective enquiry, but this has important resource implications for stretched emergency department settings, which have not yet been researched. The implications of any additional workload (including onward referrals and multi-agency working) on consultation-psychiatric liaison services warrant consideration. The most recent published information on the resourcing of consultation-liaison psychiatry services in the acute setting from LP-MAESTRO reports that the provision of services remains suboptimal, with 79% of services not reaching the minimum standards for resourcing in England.^10,11^ A similar pattern has been observed internationally.^12,13^ This is reflected in the findings of Knipe et al, who identified professionals feeling that they do not always enquire deeply enough because of limited time during consultation-psychiatric liaison assessments. On the other hand, identifying domestic abuse as a primary stressor in a presentation of self-harm and then acting to reduce further domestic abuse, perhaps by involving advocacy and safeguarding, could reduce further distress, self-harm/suicide and emergency department use, suggesting that improvements in domestic abuse responses would increase service quality.

Knipe and colleagues found that professionals need access to resources and time to respond appropriately to domestic abuse (‘space for action’). There remains much to be explored about how professionals see their own interventions and responses, for example risk assessments, practical support and open questioning. For example, taking a comprehensive history, including gathering good-quality information on who is in the household or family, especially children, can help inform risk assessments and the interface with other agencies, and can promote the safety of victims and children as part of a coordinated response. Perhaps ‘action’ should be seen as a broader suite of responses (rather than just prescribing, detention, referral to another mental health team, etc.). One important action in responding to a person exposed to domestic abuse or perpetrating domestic abuse is to acknowledge the harm that has been caused by the perpetrator’s behaviour. In this context, using the terminology of domestic abuse can help support decision-making, for example, for a perpetrator to access support for their behaviour and risk factors (such as alcohol misuse).

Knipe and colleagues investigated professional responses to domestic abuse and self-harm in depth. Strengths of the study lie in the use of a relatively narrowly defined professional group and authors from a range of disciplinary perspectives, with the paper indicating considerable reflection on the participants’ accounts via a formal qualitative method (reflexive thematic analysis). On the other hand, the focus on specific multidisciplinary settings might make it more difficult to identify themes pertaining to particular professional backgrounds (e.g. doctors as opposed to nursing staff).

Qualitative research with professionals working in mental health services offers certain practical advantages to addressing self-harm care. However, it is not without challenges: asking experienced professionals to detail their professional approaches can sometimes allow the possibility of disclosing unsafe practice, although the literature does not indicate that this has ever happened. Professionals can be afraid to speak about problems in the practice of their peers, or of their own practice. Generalising these results to policy is challenging and might be addressed by studying professional responses with larger-scale approaches such as surveys.

Knipe and colleagues interviewed professionals about their experiences of assessing domestic abuse, which the authors say referred to responses to both victims and perpetrators. Based on the report, it seems nearly all analysed accounts referred to victim/survivors, rather than perpetrators, of domestic abuse. This is understandable and even unsurprising, given that healthcare professionals often report greater lack of knowledge in responding to perpetrators than victims of domestic abuse. Nevertheless, observational evidence suggests strong associations between self-harm and perpetration of intimate partner violence (IPV), including self-reported perpetration of partner violence. The prevalence of IPV perpetration in emergency department self-harm presentations may be somewhat lower than that for partner violence victimisation, and the clinical contexts may be somewhat distinct, especially in terms of gender. The assessment of possible IPV perpetration in the emergency department context presents significant research and quality improvement challenges, but there is evidence that perpetrators of IPV are willing to discuss their harmful behaviour when asked sensitively. However, where these conversations occur without the clinician having the capacity or knowledge to respond appropriately, they may be associated with risks including collusion, worsening the situation (e.g. by raising perpetration behaviour in an assessment with a couple), and medico-legal risks. These risks indicate a need for coordinated action, encompassing the development of training, guidelines and brief behaviour change interventions in partnership with domain experts, patients/carers and clinicians. These approaches might be implemented through quality improvement mechanisms, as well as through research approaches. Given the limited data on perpetrators gathered in this study, barriers to enquiring about perpetration of IPV might be a useful focus for further study.

Knipe and colleagues’ work provides new evidence on the health service response to domestic abuse. A substantial proportion of consultation-liaison psychiatry consultations in the emergency department are not related to suicidality or self-harm; rather, they are driven by other presentations such as psychosis. Psychiatrists should recognise the possibility of family violence (a type of domestic abuse) in these presentations, especially where family members are taking on caring responsibilities. The participants in Knipe and colleagues’ study attached importance to the presence of *children* (‘If children were involved, questions about domestic abuse became more explicit and routine because of safeguarding concerns’) in history-taking. This is encouraging and offers an opportunity, as domestic abuse is a fundamental cause of childhood adversity. Asserting the message that all children growing up around domestic abuse are negatively affected by it could be a helpful motivation for improving practice in this area. Like other qualitative work with health professionals, Knipe et al identified substantial numbers of participants with their own experiences of domestic abuse as victims. Psychiatrists should be sensitive to possible experiences of domestic abuse among colleagues as well as in their patient group.

Knipe et al’s findings suggest that consultation-liaison psychiatry assessments in the emergency department could present a helpful opportunity for improving responses to domestic abuse in the health system, thereby improving service quality. More needs to be learnt about how to safely identify perpetrators of domestic abuse in the emergency department and how to address the impact of domestic abuse on children, including coercive control and other forms of non-physical abuse.

## Data Availability

Data availability is not applicable to this article as no new data were created or analysed in this study.
